# Supplementary material for: Trends and Patterns of Knee Osteoarthritis in China: A Longitudinal Study of 17.7 Million Adults from 2008 to 2017
Source: Int J Environ Res Public Health. 2021 Aug 23;18(16):8864. doi: 10.3390/ijerph18168864 (PMC8395063; doi:10.3390/ijerph18168864)
Supplement: Supplementary file 1 [file ijerph-18-08864-s001.zip › ijerph-1326742-supplementary.pdf]

Supplementary Table S1. Crude prevalence and incidence of diagnosed knee osteoarthritis in Beijing, China, 2008–2017.

	2008	2009	2010	2011	2012	2013	2014	2015	2016	2017
Prevalence (%, 95% CI)										
Overall	1.384	1.767	2.649	3.477	3.883	3.853	4.625	4.963	4.497	4.639
	(1.377,1.392)	(1.759,1.775)	(2.640,2.658)	(3.468,3.486)	(3.874,3.892)	(3.844,3.861)	(4.616,4.635)	(4.954,4.972)	(4.449,4.506)	(4.631,4.648)
Men	0.796	1.034	1.605	2.139	2.405	2.440	3.066	3.364	3.071	3.213
	(0.788,0.803)	(1.026,1.042)	(1.596,1.614)	(2.129,2.148)	(2.396,2.415)	(2.431,2.450)	(3.055,3.076)	(3.354,3.375)	(3.061,3.081)	(3.204,3.223)
Women	2.137	2.702	3.966	5.143	5.702	5.589	6.543	6.930	6.258	6.407
	(2.124,2.150)	(2.688,2.716)	(3.951,3.982)	(5.126,5.159)	(5.686,5.719)	(5.573,5.637)	(6.526,6.560)	(6.914,6.947)	(6.243,6.274)	(6.392,6.422)
Incidence (per 1000 person-years, 95% CI)										
Overall	12.62	15.75	16.34	21.48	21.50	18.45	22.69	18.83	15.37	14.41
	(12.55,12.69)	(15.67,15.82)	(16.27,16.41)	(21.40,21.56)	(21.43,21.57)	(18.38,18.51)	(22.62,22.76)	(18.76,19.21)	(15.32,15.43)	(14.37,14.46)
Men	7.31	9.63	10.43	14.00	14.38	13.06	17.00	14.49	11.88	11.29
	(7.24,7.38)	(9.55,9.70)	(10.35,10.50)	(13.92,14.08)	(14.30,14.45)	(12.98,13.13)	(16.92,17.08)	(14.41,14.56)	(11.81,11.95)	(11.23,11.36)
Women	19.41	23.65	23.99	31.13	30.72	25.49	30.19	24.60	20.07	18.64
	(19.28,19.53)	(23.51,23.79)	(23.86,24.11)	(31.00,31.27)	(30.59,30.84)	(25.37,25.61)	(30.07,30.31)	(24.49,24.71)	(19.97,20.17)	(18.55,18.73)
